# Reburial potential and survivability of the striped venus clam (*Chamelea gallina*) in hydraulic dredge fisheries

**DOI:** 10.1038/s41598-021-88542-8

**Published:** 2021-04-27

**Authors:** Giada Bargione, Andrea Petetta, Claudio Vasapollo, Massimo Virgili, Alessandro Lucchetti

**Affiliations:** 1 Department of Biological, Geological and Environmental Sciences, University of Bologna, Piazza di Porta San Donato 1, 40126 Bologna, Italy; 2 Institute for Biological Resources and Marine Biotechnologies (IRBIM) of Ancona (Italy), National Research Council (CNR), Largo Fiera della Pesca, 1, 60125 Ancona, Italy

**Keywords:** Zoology, Ecology, Environmental sciences, Environmental social sciences

## Abstract

The striped venus clam (*Chamelea gallina*) is the main edible bivalve living in Italian waters. According to Regulation (EU) 2020/2237, undersized specimens (total length of the shell, < 22 mm) must be returned to the sea. *C. gallina* specimens of different size classes that had undergone hydraulic dredging and mechanized sorting were analysed for reburial ability in a laboratory tank and for survivability in the laboratory (135 clams, 21 days) and at sea (320 clams, 15 days). In the tank experiments, the reburial times (T_50_ and T_90_) and the upper (+) and lower (−) confidence intervals (CIs) of the whole sample were about 4 h (CI+ 4.4, CI− 3.6) and 8 h (CI+ 8.2, CI− 7.7), respectively, and were significantly shorter for the medium-sized clams (22–24.9 mm) than for the smallest (< 21.9 mm) and the largest (> 25 mm) specimens. For the field survivability experiments, clams under and above the minimum conservation reference size were placed in separate metal cages. Survival rates were 94.8% and 96.2% respectively in the laboratory and at sea, without significant differences between the two experiments or among size classes. These findings conclusively demonstrate that *C. gallina* specimens returned to the sea have a very high survival probability and that they can contribute to mitigate the overexploitation of natural populations.

## Introduction

The striped venus clam (*Chamelea gallina* Linnaeus, 1758), an edible and commercially valuable bivalve, is an infaunal filter-feeding clam of the family Veneridae. It is widespread in the Mediterranean and the Black Sea and along the eastern Atlantic coasts, where it inhabits the fine well-sorted sand biocoenosis described by Pérès and Picard^1^. It thrives in sandy and muddy–sandy sediments and tolerates narrow salinity and temperature variations^2^. In particular, a study of its presence as a function of sediment type, conducted in the north-western Adriatic Sea near Venice^3^, has reported that *C. gallina* does not inhabit substrata with a redox potential lower than + 50 mV (i.e. it does not tolerate reduced conditions) or with a sand fraction lower than 90% (as it does not tolerate anoxic conditions). Accordingly, the species is concentrated in limited areas 0–12 m in depth and up to 1–2 nautical miles (nm) from the coastline^4^. Its high abundance in the central and northern Adriatic Sea is determined by the large amount of nutrients, particles and organic matter which are supplied by the massive outflow of the river Po and by the coastal flows, which are carried along the Italian Adriatic coast by the Western Adriatic Current^5,6^.

In Italy, this bivalve fishery involves 636 hydraulic dredgers that provide about 1500 jobs; its annual landings of ≈ 10,000 metric tonnes are worth about €51 million^7^. Since the 1970s, technological innovations such as hydraulic dredges and mechanized sorting equipment have considerably increased the fishing effort and exploitation level of *C. gallina* beds in the northern Adriatic Sea, resulting in overexploitation in some areas^8^. Another consequence has been the loss of the largest specimens (> 25 mm in shell total length; TL), owing to the efficiency and size selectivity of the gear, which has been estimated to catch nearly 100% of commercial-sized clams^4^, as well as to inadequate stock management and protection measures^9^. On the other hand, monitoring surveys performed in the past 20 years have detected a massive amount of juveniles, with catches of undersized specimens exceeding 90% in 2016^10^. Because the species reaches the size at maturity at 15–17 mm TL in the first year of life (Atlantic Ocean^11^, Marmara Sea^12^, Adriatic Sea^13^), in Italian territorial waters the Minimum Conservation Reference Size (MCRS) of 25 mm TL (Regulation (EC) 1967/2006^14^) has been reduced to 22 mm TL (Delegated Regulation (EU) 2016/2376^15^, Regulation (EU) 2020/3^16^, and Regulation (EU) 2020/2237^17^). Notably, according to Regulation (EU) 1380/2013^18^, the obligation of landing all specimens under the MCRS does not apply to “species for which scientific evidence demonstrates high survival rates, taking into account the characteristics of the gear, of the fishing practices and of the ecosystem”; in such cases, fishers are required to return undersized specimens to the sea immediately after sorting.

Whereas gear efficiency has been studied extensively (e.g. Refs.^19,20^), data on the effects of fishing on population sustainability are more limited^8,9,21,22^. Clams harvested with hydraulic dredges are hauled up from the seabed, dumped into a collecting box on board and conveyed to a mechanized sieve for sorting. Since the smaller specimens that pass through the sieve are returned to the sea through a waste exhaust pipe, discarded clams undergo considerable physical stress^23^. Even though discards are believed to mitigate the overexploitation of natural populations, the mechanical stress to which they are subject has the potential to reduce their survivability^22,24^.

The survivability of the striped venus clam (e.g. Refs.^2,22,25^) and other bivalve species (e.g. Ref.^26^) has largely been studied in terms of the natural ability of bivalves to survive periods of aerial exposure^26^. The present study is the first attempt to assess the survivability of *C. gallina*, (a) by reproducing as closely as possible the sea habitat conditions in the laboratory and (b) through field tests in the natural environment. The possible differences in reburying and survivability capacity across sizes were examined by studying undersized individuals (discards) as well as commercial-sized specimens.

## Materials and methods

### Gear characteristics and sample collection

**Figure 1:**
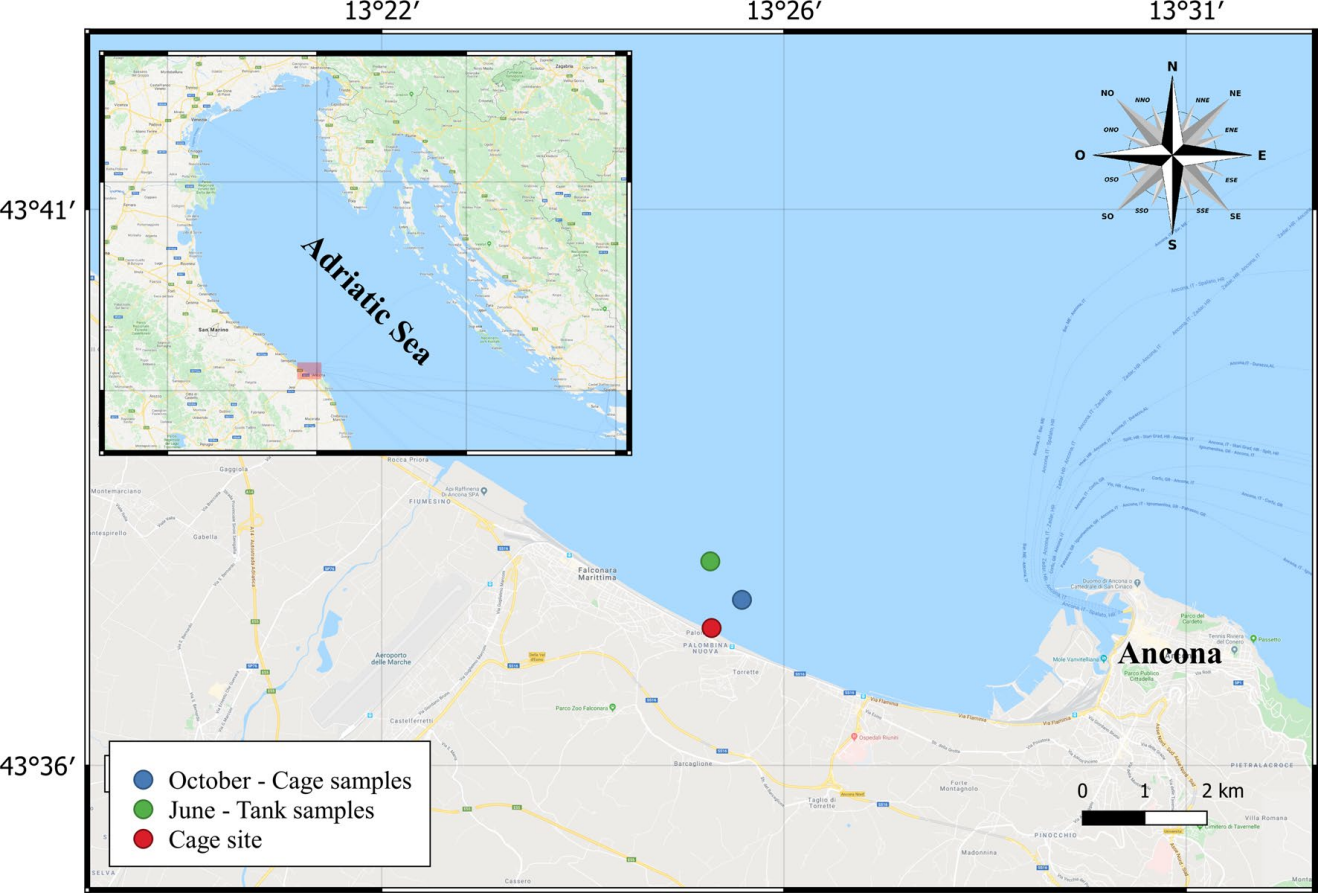
Map of the sampling and field experiment areas generated through the QGIS software version 3.10.10 (www.qgis.org) by one of the co-authors (AP).

Clams were harvested by a commercial hydraulic dredger (LOA, 15.8 m; tonnage, 9.7 GT; engine power, 110 kW) using standard gear and sorting methods in two fishing trips carried out in the Ancona Maritime District (central Adriatic Sea, Fig. 1). Dredging was conducted at ≈ 3 m depth 0.3 nm off Ancona on a fishing ground characterized by fine, well-sorted sandy bottoms. The tank experiments were performed in June 2019 and the experiments in the sea in October 2019.

The hydraulic dredge used for this study consists of a metal cage 2.8 m wide whose bottom is made of metal rods placed at 12 mm intervals to retain the clams. A blade is fitted at the dredge mouth to cut the soft bottom, whereas a hose connected to a centrifugal water pump ejects pressurized seawater from nozzles to fluidize the sediments. The cage is mounted on two sledge runners to prevent it from digging into the sediment. When the cage is hauled up, the catch is dumped onto a collecting box and conveyed to a mechanized vibrating sieve for sorting. The sieve consists of 4 stacked sorting grids with hole diameters decreasing from 32.5 to 20.3 mm (see Sala et al.^20^ for details). The specimens used in the present experiments were collected directly from the vibrating sieve (≥ MCRS) or from the waste exhaust pipe (< MCRS). Clams of all sizes were used for the experiments, provided that they were intact (i.e. without shell damage, scratches, chipped edges or crushed umbos). To mimic their being returned to the sea after sorting while minimizing the stress induced by aerial exposure, all the clams used for the survival experiments were immediately placed into a plastic tank containing seawater collected during the fishing hauls. To avoid further stress, the plastic tank was handled gently until the clams were placed in the laboratory tanks/sea cages.

To reproduce the clams’ environmental conditions in the laboratory, seawater temperature and salinity were recorded during fishing operations using a CTD probe (model YSI 30).

### Laboratory experiments

Clam reburying capacity and survivability under controlled conditions were assessed following the guidelines established in the Workshop on Methods for Estimating Discard Survival (WKMEDS—ICES, 2015).

**Figure 2:**
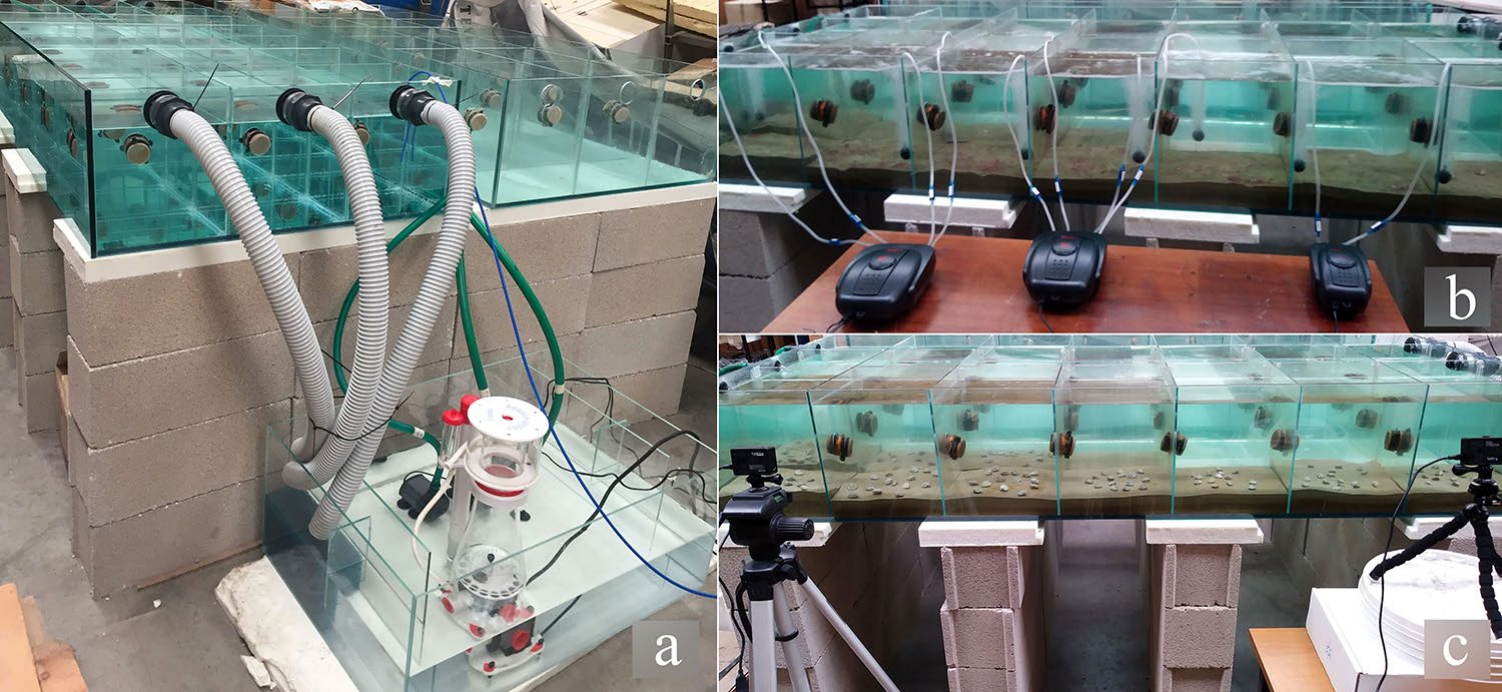
Experimental glass tank for clam reburial and survival experiments. (**a**) The sump (centre) was connected to the tank through 3 pumps, forming a closed circuit. The sump was equipped with sponges serving as mechanical and biological filters (left) and with a skimmer system to remove the organic particulate matter (middle). (**b**) Aerators placed outside the tank (picture taken on day 15). No clams are visible on the sediment surface, since by 21 h all had reburied. (**c**) GoPro cameras placed outside the tank and programmed to shoot at 15-s intervals. At the beginning of the reburial experiment, all clams are still visible on the sediment surface.

The captivity study used a glass tank divided into 9 communicating sub-compartments, each measuring 30 × 30 × 35 cm, connected to a sump (180 l, 90 × 60 × 35 cm), forming a closed system (Fig. 2a). The tank was filled with osmotic water—obtained by a purification process using a partially permeable membrane system to remove ions, larger particles and unwanted molecules—added with salt to achieve a salinity of 35 ppm; water temperature was set at 20.0 ± 1 °C and maintained by a cooler connected to the sump through a pump with a flow rate of 1500 l/h. Constant temperature, salinity and dissolved oxygen were ensured throughout the experiment. Aeration was provided by 3 aerators (8 W, 550 l/h) through silicon tubes ending with an air stone (Fig. 2b). Water quality, i.e. ammonia, nitrate, nitrite, and phosphate concentrations and pH, was measured with reagent tests (SERA or Jbl) at weekly intervals, to exclude stress due to non-optimal or sub-toxic conditions. About 7 cm of sand collected from the harvesting area (43.6198 N; 13.4252 E) was placed on the bottom of each sub-compartment, after sieving to remove shell fragments and benthic macrofauna (e.g. bivalves, gastropods, crustaceans, echinoderms), thus avoiding clam overestimation and potential predation. Water recirculation was ensured by 3 pumps (flow rate, 950 l/h) installed in the sump, with the water flowing from the tank into the sump by gravity, falling on sponges that served as mechanical and biological filters. The filter was previously matured by adding 10 vials (each 1 ml) of nitrogen cycle bacteria one week before beginning the experiment. A skimmer system (flow rate, 520 l/h) was installed in the sump to remove organic particulate matter. Clams were fed daily ad libitum with marine gel phytoplankton (Easy booster 25) consisting of 31% *Nannochloropsis*, 33% *Isochrysis*, 18% *Tetraselmis* and 18% *Phaeodactylum*.

For the tank experiments, clams were divided into 3 size classes: 1, 19.0–21.9 mm TL; 2, 22.0–24.9 mm TL; and 3, 25.0–27.9 mm TL. A total of 15 specimens with 3 replicates per size class were placed in each sub-compartment.

#### Reburying capacity

Reburial ability was assessed by time-lapse monitoring (shots taken at 15-s intervals) using two GoPro 5 Black cameras positioned outside the tank (Fig. 2c). When clams were no longer visible on the sediment surface the cameras were switched off. The time required for clams to become invisible was estimated using shots taken at 30-min intervals, the number of clams still visible in each shot being counted and recorded. Data were processed separately for each size class.

#### Survivability in captivity

The laboratory survivability experiment lasted 21 days. In the morning and late afternoon the tank was examined for dead specimens (clams with open valves), which were removed and measured. At the end of the experiment, the surviving clams were extracted from the sand, counted and measured. The percentage of clams under and above the MCRS was calculated and compared.

### Survivability at sea

The survivability experiment at sea lasted 15 days and was performed off Palombina beach, near the clam beds (Fig. 1). Two metal cages (100 × 120 × 40 cm) marked on the surface by floats were anchored to the bottom at ≈ 1 m depth. The cage surfaces were covered with netting: the bottom panel had a nominal mesh size of 5 mm, whereas the wider mesh size of the other panels (20 mm) ensured water circulation and prevented predation. The clams, 160 specimens < MCRS and 160 ≥ MCRS (Supplementary Video 1), were placed in the respective cages through an opening on the top and left undisturbed for 15 days to avoid further stress. Dead specimens were counted at the end of the experiment.

### Data treatment and statistical analysis

A generalized linear model (GLM) with a binomial distribution was applied to analyse clam reburial time and survivability. The factors for the former analysis comprised time (continuous variable) and size class (3 levels); their interaction, indicating a significant difference among factor levels, was also investigated^27^. Model selection was based on Akaike’s Information Criterion (AIC). The log-likelihood ratio test (based on χ^2^ distribution) was used to assess factor significance in the model. Whenever a factor was significant, a Wald z-test based on χ^2^ distribution was applied to determine the significance of pairwise estimates^28^. After model selection, over-dispersion and residuals were analysed to further validate the selected model.

For the reburial study, the time when none of the clams were still visible on the sediment surface was recorded; the times at which 50% (T_50_) and 90% (T_90_) of the specimens were likely reburied and their upper (+) and lower (−) 95% Confidence Intervals (CIs) were computed both for the whole sample and for the 3 size classes.

For the survivability study, the proportions of survivors under and above the MCRS at the end of the trials were calculated for the laboratory and the field experiments. Moreover, to compare survivability as a function of the MCRS (< 22 and ≥ 22 mm TL), the GLM considered the “Experiments" (Sea and Laboratory tests) and the “Size Classes” (under and above MCRS) as two-level factors. Condition (2 levels: number of live and dead individuals) was used as a response variable and the whole dataset was treated as a contingency table^27^. All analyses were performed using the *stats* package of the freely available software R (version 3.6)^29^.

## Results

### Reburying capacity in the tank

**Figure 3:**
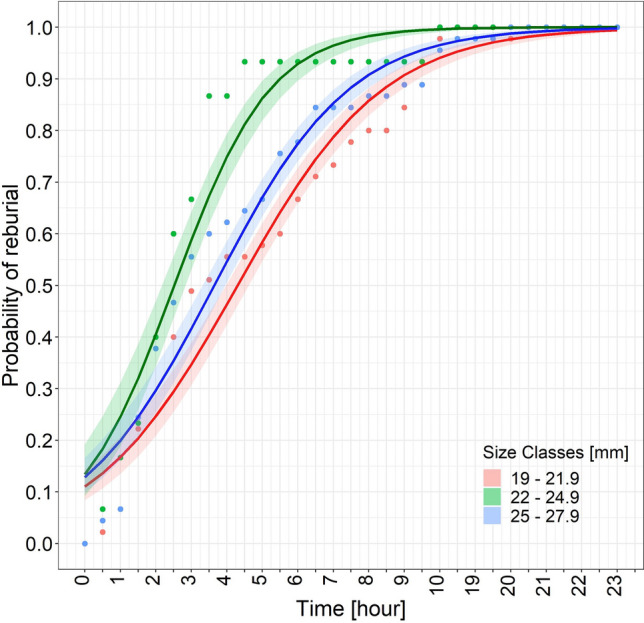
Logistic curves of the reburial probability calculated for the three size classes. Coloured areas around the lines: confidence intervals of the theoretical model; points: experimental observations.

By 21 h, all specimens had reburied regardless of their size (Supplementary Video 2). However, the χ^2^ test highlighted a significantly different (*p* < 0.01) reburial ability depending on size class (Table 1). The Wald z-test detected a significant difference between size classes 1 and 2 (*p* < 0.01) and 2 and 3 (*p* < 0.01), but not between classes 1 and 3 (*p* = 0.32) (Table 2). Medium-sized clams were the fastest to rebury (Fig. 3); their T_50_ was 3.0 (CI+ 3.4, CI− 2.7) and their T_90_ was 6.0 (CI+ 6.3, CI− 5.8). The T_50_ of the smallest and the largest clams was 4.8 (CI+ 5.3, CI− 4.5) and 4.1 (CI+ 4.5, CI− 3.8), respectively, whereas their T_90_ was 9.4 (CI+ 9.7, CI− 9.2) and 8.4 (CI+ 8.7, CI− 8.2), respectively. The T_50_ and T_90_ for the whole sample (135 clams) were ≈ 4 h (CI+ 4.4, CI− 3.6) and 8 h (CI+ 8.2, CI− 7.7), respectively.

**Table 1:** Log-likelihood ratio test showing significant differences in reburial ability for each size class.

Effects	df	Deviance	AIC	LRT	*p* (> χ^2^)
**Log-likelihood ratio test**					
Time	1	1945.7	2170.3	1774.1	< 0.0001
Size class	2	244.7	467.2	73.1	< 0.0001
Time × size class	2	171.6	398.2	18.3	0.0001
Full model		153.3	383.2

**Table 2:** Wald z-test showing significant differences in reburial time in size class 1 versus 2 and size class 2 versus 3.

Pairwise interactions	df	χ^2^	*p*
**Wald z-test**			
1 versus 2	1	15.3	< 0.0001
1 versus 3	1	0.98	0.32
2 versus 3	1	10.4	0.0012

### Survivability experiments in the tank and at sea

**Figure 4:**
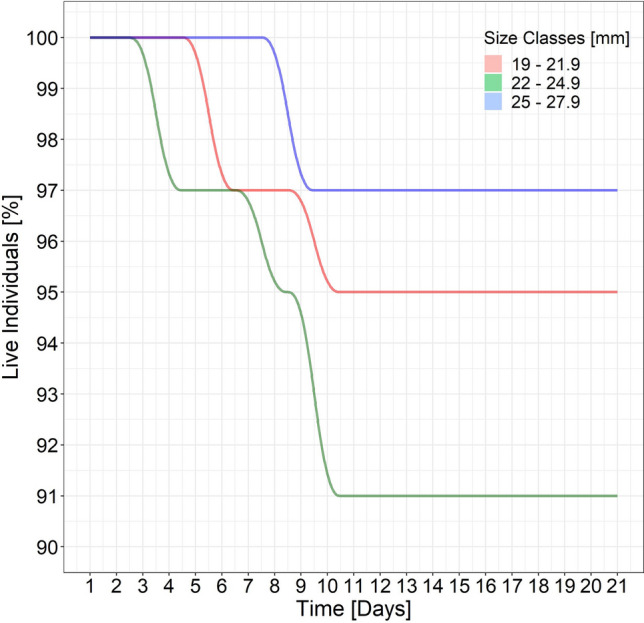
Percentage of clams of each size class surviving during the tank experiments.

By the 21st day in the laboratory tank, 7 of the 135 specimens (2 < MCRS and 5 ≥ MCRS) had resurfaced and died. Deaths were recorded from day 4 to day 10 and showed no size dependence (Fig. 4). The survival rates of commercial-sized and undersized specimens were respectively 94.4% and 95.5% (mean, 94.8%).

By the end of the 15th day in the sea cages, 12 of the 320 specimens (4 < MCRS and 8 ≥ MCRS) had died. The survival rate of the commercial-sized and the undersized specimens was respectively 95.0% and 97.1% (mean, 96.2%), again without any size dependence.

According to the χ^2^ test, mortality in the tank and field experiments and among size classes was not significantly different (*p* = 0.90) (Table 3).

**Table3:** Log-likelihood ratio test showing the absence of significant differences between survival in the laboratory and at sea and between size classes.

Effects	df	Deviance	AIC	LRT	*p* (> χ2)
**Log-likelihood ratio test**					
Experiments	1	0.3	17.5	0.3	0.583
Size class	1	0.4	17.6	0.4	0.526
Experiments × size class	1	0.1	19.2	0.1	0.904
Full model		0.1	21.1		

## Discussion

Despite the economic importance of *C. gallina*, data on the reburial ability and survival of discarded clams returned to the sea are scarce. A tank study of reburial ability by Morello et al.^30^ reported a T_50_ of about 3 h, which is very similar to the one (T_50_ < 4 h) estimated in the present study for the whole sample (135 clams); even when reburial time was calculated separately for the three size classes, the T_50_ ranged from 3 to 4.8 h. Morello et al.^30^ also found that less than 35% of clams were still visible after 4 h, whereas by 8 h, 90% of our sample had reburied and by 21 h no clams were visible any longer. Differences in reburial time may be due to the different energy stores of specimens of different size classes. Indeed, Moschino and Marin^2^ have reported that larger clams store more energy whereas smaller clams consume more energy per unit of volume. Accordingly, even though larger clams should rebury faster because of their larger energy stores, they also have a larger surface area to be reburied, whereas smaller specimens have a smaller surface area to rebury, but less stored energy per unit of volume. These considerations may explain the absence of significant differences in reburial time between the larger (size class 3) and the smaller clams (size class 1) of our sample. These data suggest that medium-sized clams (size class 2) may have a more favourable balance between surface area and stored energy, since they reburied significantly faster than the other size classes. Henderson and Richardson^31^ have sought potential relationships between shell size and the time required to rebury in other bivalve species (*Ensis siliqua* and *Ensis ensis*), using time-lapse video to analyse burying behaviour in different fine and coarse sediment types. They found a relationship only for *E. siliqua* in fine sediment (smaller individuals reburied comparatively faster). This suggests that shell shape may play an important role in burying time in relation to specimen size; notably, in some cases the elongated shell of the smaller razor clams may provide an advantage on the more globous shell of the smaller striped venus clams. Bivalve reburial ability has also been studied in situ following dredging. For example, Chícharo et al.^32^ tested the reburial time on the seabed of *Spisula solida* specimens dislodged by the dredge or hand-collected by divers. By 12 min, all the hand-collected specimens had reburied, whereas those that had been dislodged by the dredge took more than 30 min to rebury completely. Leitão et al.^33^ tested the burying response of discarded undersized cockles (*Cerastoderma edule*) that had been hand-dredged or harvested with a knife; they found that 83% of specimens had reburied within 15 min irrespective of the collection method, whereas only 10% were still visible on the sediment surface 1 h after being discarded. In an underwater study comparing a traditional dredge to an innovative dredge for *Callista chione*^34^, the macrobenthic species that had escaped through the metal rods of the new dredge, which included bivalves with and without commercial value (*C. chione, Pharus legumen, E. ensis, Solen marginatus, E. siliqua, Mactra glauca, Lutraria anguistor, Laevicardium crassum, S. solida, Venus striatula, Dosinia exoleta*), reburied soon after they escaped. These studies describe a relatively faster reburial ability of bivalves tested directly at sea or replaced on the bottom soon after dredging compared to those transferred into containment facilities (present study and Morello et al.^30^). This observation may lead to even more reassuring considerations on the reburial ability of undersized *C. gallina* specimens discarded directly at sea during commercial fishing operations. However, aerial exposure exceeding 1 h has been reported to involve a significant reduction of reburial ability and of the physiological response to dredging-induced stress in *S. solida*^35^*.*

Clam mortality in our laboratory experiments was low (≈ 5%) and did not correlate with shell size, whereas other studies have found that the smallest clams are more likely to die^36,37^. Moreover, at variance with the finding that clams may be more likely to die immediately after being placed in the tanks or around the end of experiments due to containment^38^, the mortality of our captive *C. gallina* specimens was not related to a particular time. In our study, neither the harvesting and sieving process nor captivity in the tank induced significant mortality, suggesting that other factors (e.g. disease, parasites) may have caused the death of weaker or less healthy specimens.

Although Breen et al.^39^ recommend monitoring the key environmental parameters (e.g. depth, temperature, salinity) during captivity, the high survival rate of our specimens suggests that the slight depth difference (1–1.5 m) between the fishing ground and the cage site did not affect survivability. Similarly, specimen size did not affect survivability, since only 7 individuals died in captivity (2 < MCRS and 5 ≥ MCRS) and 12 individuals died in the sea trials (4 < MCRS and 8 ≥ MCRS).

This was the first study investigating the survival of discarded striped venus clams in environmental conditions mimicking the natural habitat. The similar mortality recorded in the laboratory and the field experiments demonstrates the ability of our conditions in captivity to closely mimic those at sea. Studies of clam survival in relation to aerial exposure have found L_50_ values of 4 days^25^, 5–6 days^2^ and 6.2 days^34^. The season, together with other biotic (e.g. gonadal development and energy storage) and abiotic factors (e.g. seawater temperature and salinity), influences clam conditions^22,40^ hence survivability in air. A study of survival in air of *Mytilus edulis* from the Dutch coast^26^ has found that pollutants accumulated in clam tissue reduce survival time in air. Exposure to different pollutant concentrations for different times inhibited bivalve reburial ability, leading to death (e.g. Refs.^41,42^).

Another stress factor that influences the survival potential and condition of captured clams is the dredging-fishing effort^21,43^. Clam beds are subject to extremely high fishing pressure, as demonstrated by the Side Scan Sonar surveys in the Adriatic Sea^44^, and to high discard rates^19,23^. Notably, Petetta et al.^19^ have estimated that the first size selection performed by the dredge on the seabed does not spare undersized individuals, since more than 58% of the clams caught are under the former MCRS of 25 mm TL and undergo sieving, which retain less than 5% of undersized individuals^20^. Mechanical sorting and discarding into the sea may cause a physiological stress and physical damage to small clams, which may be harvested as many as 20 times a year^8,21^.

Moschino et al.^22^ have examined the effect of hydraulic dredging on the physiological response of *C. gallina* from the north-western Adriatic Sea, both in the laboratory and at sea. In laboratory experiments, mechanical stress was simulated by vortexing the clams in a mixer, whereas field experiments included four levels of stress, the lowest involving manual sampling by scuba divers and the highest involving exposure to high water pressure and mechanized sorting, mimicking collection by commercial gears. The laboratory specimens showed a lower physiological response than controls and a shorter survival in air (L_50_, 6 days vs. 10 days), whereas those undergoing the sea trials exhibited a declining physiological response and survival in air (L_50_, ≈ 5 days) as the stress level increased. At variance with these findings, our clam sample exposed to high water pressure and mechanized sorting showed very high survival rates, also considering the additional stress due to handling and transport to the tank or the sea cages. A study of mortality related to hydraulic dredging^24^ has reported a rate of 2 to 20% (mean, ≈ 10%) corresponding to a survival rate of at least 80%. Considering that the water pressure used in the study was higher than the regulation 1.8 bar (DM 22/12/2000^45^), the mortality rate using the legal water pressure should be lower. A 7-day captivity study has assessed the survivability of three undersized commercial bivalve species (*Donax trunculus*, *S. solida* and *C. gallina*) harvested with hydraulic dredgers without recreating the natural sea bottom habitat. Undersized and commercial-size individuals of the three species were divided into those with intact shells and shells with the edge chipped. At the end of the experiments, the survival rate of the intact specimens ranged from 86 to 100% irrespective of species and size, in line with the survival rate of the undamaged clams analysed in our study. The survival rate of the chipped specimens ranged from 24.2 to 60%^46^, suggesting the need for additional work on the survivability of damaged individuals.

Altogether, previous findings and the present data—documenting that a very large proportion of clams survive harvesting and sorting and that they show a high reburial ability and survival rate after reburying—demonstrate the high survival potential of *C. gallina* and support the claim that undersized specimens of this bivalve can be returned to the sea *as per* Regulation (EU) 2020/2237^17^. The present data suggests that a very high proportion of discarded *C. gallina* survive and grow to the commercial size (MCRS), which is reached on around 2 years of age^47^. The common observation of clams with repaired shells further testifies to their survival ability. Longer-term studies are clearly needed to understand the extent of the ecological disruption induced by dredge-fishing and discarding on the feeding, growth and reproduction of discarded specimens. Further work is also required to improve our understanding of the impact of fishing gears on damaged clams if a more rational management of this important resource is to be achieved.

## Supplementary information

Supplementary Video 1.

Supplementary Video 2.

Supplementary material 3 (DOCX 14 kb)

## Data Availability

The datasets generated and/or analysed during the study are available from the corresponding author upon reasonable request.
