# Supplementary material for: Reburial potential and survivability of the striped venus clam (Chamelea gallina) in hydraulic dredge fisheries
Source: Sci Rep. 2021 Apr 27;11:9109. doi: 10.1038/s41598-021-88542-8 (PMC8079708; doi:10.1038/s41598-021-88542-8)
Supplement: Supplementary file 3 — Supplementary material 3 (DOCX 14 kb) [file 41598_2021_88542_MOESM3_ESM.docx]

**Supplementary material**

The following supplementary materials are available at *Scientific Reports* online. *Supplementary Video 1* shows clams introduced in a cage at sea, while *Supplementary Video 2* shows clams reburying inside the glass tank across time.
